# Hemodialysis water reuse within a circular economy approach. What can we add to current knowledge? A point of view

**DOI:** 10.1007/s40620-024-01989-6

**Published:** 2024-06-03

**Authors:** Faissal Tarrass, Meryem Benjelloun, Giorgina Barbara Piccoli

**Affiliations:** 1 Center of Hemodialysis 2 Mars, 466 BD 2 Mars, 20460 Casablanca, Morocco; 2 Nephrology, Centre Hospitalier Le Mans, Le Mans, France

**Keywords:** Circular economy, Sustainable water use in dialysis, Climate change, Wastewater in dialysis, Hemodialysis

## Abstract

The ongoing climate change and the ecological challenges call for sustainable medicine and, in our field, sustainable kidney care. Dialysis is life-saving and resource-consuming, and high water consumption is one of the main concerns. Circular water economy, meaning reuse and recycling of water, and recovering resources can help reducing emissions and enhancing resilience to climate change. Several actions are possible including reusing reverse osmosis reject water, employable for gardening, aquaponics or even simply for toilet flushing, or in sterilization settings, reusing spent dialysate, at least for toilet flushing, but with wider use if microbiologically purified, recovering thermal energy from spent dialysate, that can probably be done with simple devices, or using phosphate-rich spent dialysate for producing fertilizers, namely struvite. All these options may be economically sound, and all help reducing the final dialysis carbon footprint. There is room for open-minded innovative approaches to improve water-related sustainability in hemodialysis, ultimately reducing ecological impact and increasing availability.

**Graphical abstract:**

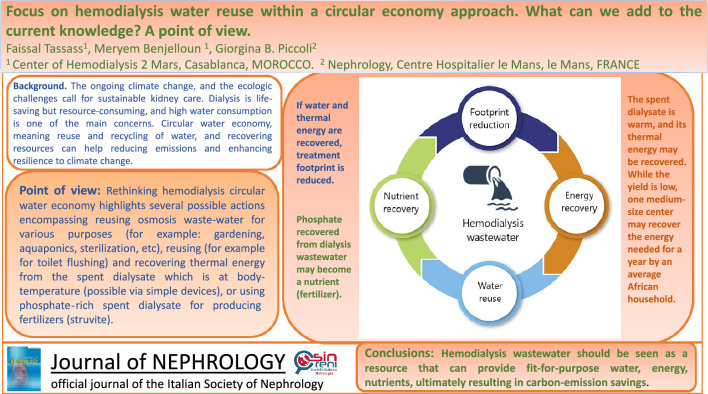

## Background

The nephrology community is becoming more and more aware that the global burden of kidney diseases, and growing demand for dialysis will drive the depletion of natural resources and increase waste production and environmental pollution, and increasingly seeks to explore ways to deliver environmentally sustainable kidney care [1, 2]. In this context, pursuing carbon neutrality [3], wastewater reuse [4], energy recovery [5] and nutrient extraction [6] have been studied.

One of the most important challenges in hemodialysis is sustainable water management. The circular water economy is an innovative approach that mimics the natural water cycle, employing reuse and recycling to turn waste into a resource [5, 6]. The hemodialysis population, currently estimated at approximately 3.75 million worldwide, consumes more than 98 million m^3^ of water per year. In the United States, over 580,000 patients are receiving hemodialysis, consuming over 18 million m^3^/year of fresh water [6]. In countries experiencing water shortages, like Morocco, where dialysis accounts for over one million m^3^ of water per year, the need to reduce the amount of water used is even more urgent [6]. While issues regarding the reduction of water usage in hemodialysis (for example: RO system selection, use of flow regulation devices, reduction of dialyste flow rate, etc.) have been widely discussed [7, 8], in this commentary we focus on some further potential strategies to promote water circularity in hemodialysis through the reuse of reverse osmosis reject water and the recycling of spent dialysate.

## Reuse of reverse osmosis reject water

**Fig. 1:**
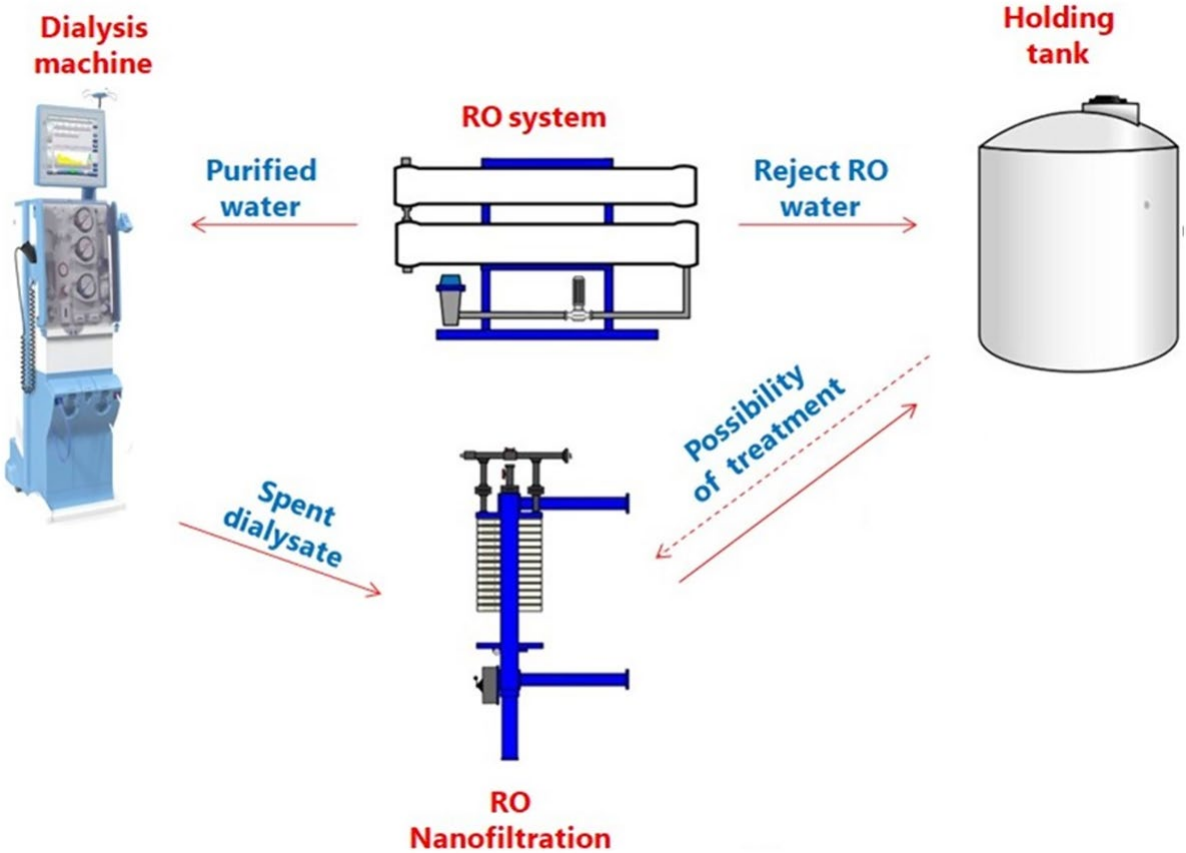
Measures for optimization of wastewater as a source of water recovery in hemodialysis

We are witnessing a rapid increase in the reuse of reverse osmosis reject water everywhere in the world. The water rejected from osmosis can be reused in gardening and farming, for mopping floors, flushing toilets, cooling, and sterilizing (Fig. 1) [6]. Before reusing reject water, the salt level should be evaluated by measuring its electrical conductivity. While standards vary slightly from one country to another, water with conductivity below 1500 µs/cm can generally be used for irrigation (for example, watering gardens, aquaponics or hydroponics), while, when conductivity is between 1500 and 2400 µs/cm it can be reused for flushing toilets or cleaning floors. Since an excessive salt level can have a negative effect on plant growth, mixing reverse osmosis reject water with water from other sources (rain, wells) allows us to dilute salts to a safe level for use in farming [4].

In a typical system for reject water reuse, water flows into a storage tank and is then redistributed. The system is regulated by float switches and excess water can be diverted to a drain [4]. Different parameters should be considered when planning a water reuse project, including the volume of reject water, its chemical composition, the location of the dialysis unit and its distance from the reuse site, the need for transmission lines and pumping requirements, storage potential and energy costs [4]. The few economic evaluations we have of reject reverse osmosis water reuse generally show profitability within a short payback time (Table 1).

**Table 1:** Case cost studies of reject reverse osmosis water reuse in hemodialysis

	Canterbury Hospital NHS—UK [9]	Countess of Chester Hospital NHS—UK [10]	Lister Hospital NHS—UK [11]	Maidland Hospital Ireland [12]	Sultan Abdul Halim Hospital Malaysia [13]
Reuse option	Toilet flushing	Toilet flushing, Laundry and other uses	Hot water	Toilet flushing	Acquaponics and horticulture
Volume of saved water	800 Liters / hour	1460 M^3^/year	3140 m^3^/ year	5240 m^3^/ year	12,000 liters/day
Implementation costs	19,000 $ (15,000 £)	14,000 $ (11,030 £)	7600 $ (6000 £)	13,100 $ (12,000 €)	$1047 (MYR 5000)
Financial savings (USD/year)	9530 $ (7500 £)	3990 $ (3143 £)	8000 $ (6300 £)	13,600 $ (12,500 €)	$524 (MYR 2500)
Payback period (months)	24	42	12	12	24

## Recycling of spent dialysis effluent

Spent dialysis effluent, which has a high saline content and is a potential source of infectious contamination, is systematically discharged. However, even this gray water is too valuable to waste and can be reused. Membrane filtration techniques, such as reverse osmosis and nanofiltration, are effective in reducing not only salt content but also an array of micropollutants and pathogens, including viruses, drugs and drug metabolites [14]. The use of reverse osmosis and nanofiltration, alone or coupled with ultrafiltration has been shown to be effective in treating spent dialysate effluent so that it can be used in gardening, with low energy consumption and a low carbon footprint, and cost savings of 20–30% in comparison to the desalination of seawater for the same use [4].

Filtration through a membrane offers significant advantages given its operational simplicity, flexibility, cost-effectiveness, reliability, low energy consumption, easy control, good stability in different operating conditions and at different temperatures, amounts of pressure and varying pH [14]. Membrane technology has been shown to be effective in the treatment of hospital wastewater for non potable uses, giving it a potential role in the circular water economy. The reuse of wastewater is especially crucial in water-scarce regions. For example, Klatt et al., suggest, depending on the effluent treatment, the reuse of treated wastewater for groundwater recharge and agricultural irrigation, and also as a cooling medium, potable water or industrial water [14].

## Energy recovery

Hemodialysis wastewater is discharged to a sewer at a temperature ranging from 20 to 25 °C, which means that it maintains considerable thermal energy [5]. We have previously estimated that up to 1600 GWh per year of thermal energy is lost in dialysis units worldwide. In other words, the discharged energy could heat approximately 140,000 homes in a European country for one year, with a potential annual cost savings of 118 million euros [5].

**Fig. 2:**
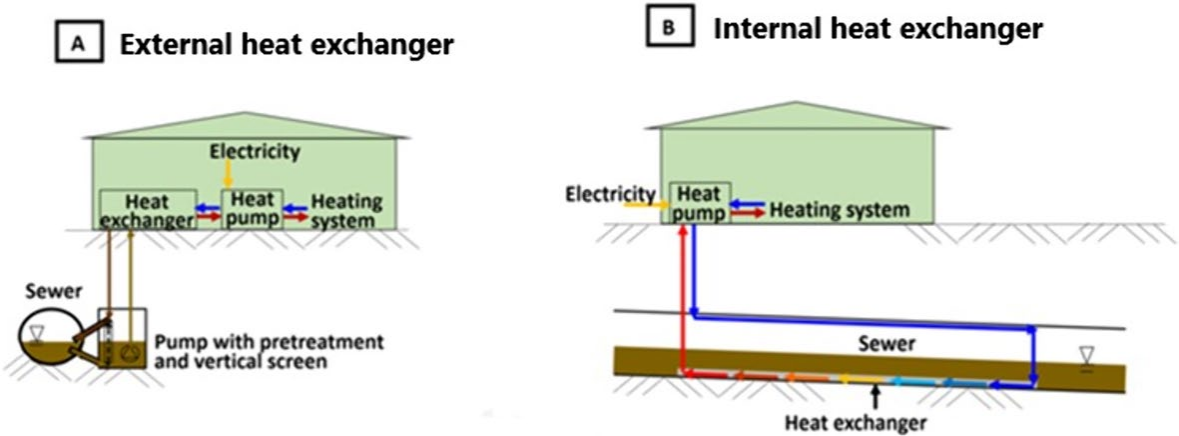
Thermal energy recovery: **A** outside a sewer and **B** inside a sewer [16]

There are two possible ways to recover heat from hemodialysis wastewater. The first is to use a heat exchanger installed in the pipe bed of the hemodialysis sewage network. The heat exchanger captures thermal energy from the wastewater, and a heat pump is used to transfer this energy to a centralized heating system. The second option is to install an external heat exchanger above ground level. The wastewater is pumped through the above-ground heat exchanger, and then returned to the sewer [15, 16] (Fig. 2).

Which option should be chosen depends on a range of factors including existing infrastructure, costs, and the needs of the hemodialysis facility. A feasibility study can help to identify the most suitable solution in a given setting. Additionally, local regulations should be taken into account when implementing such systems [15].

**Fig. 3:**
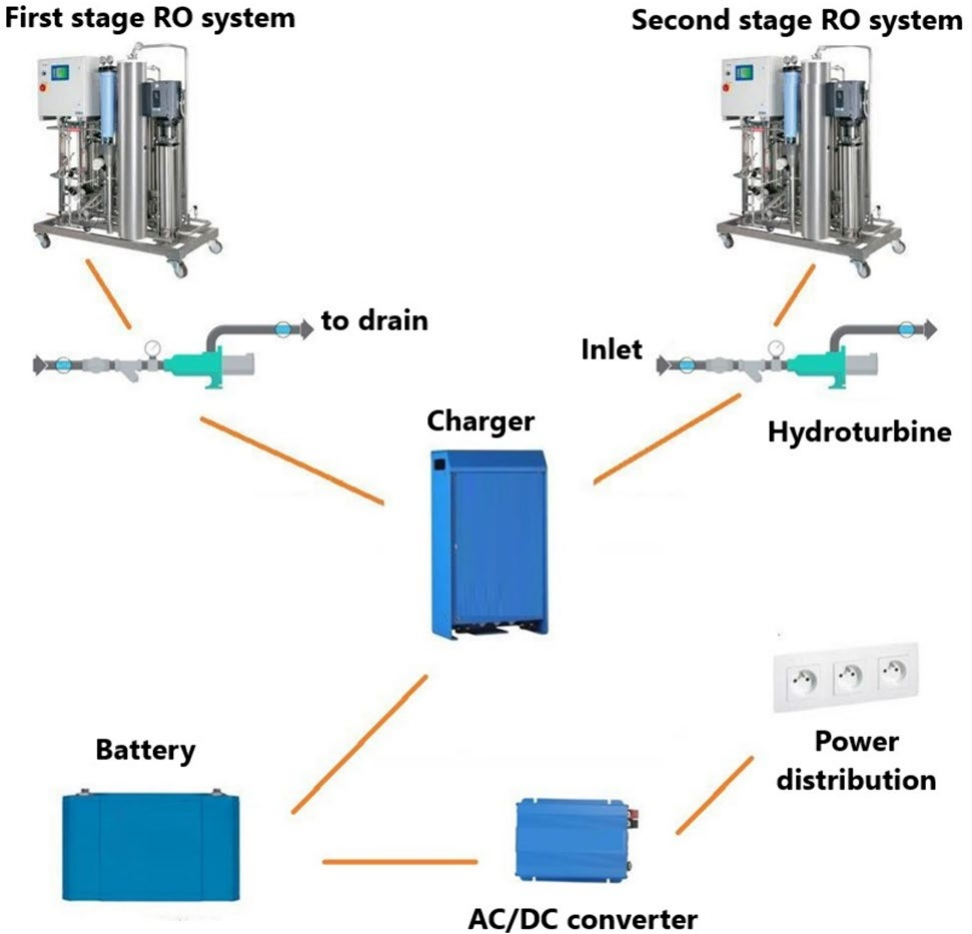
System designed for hydropower generation from hemodialysis reject RO water [17]

Hydro-electric energy can be recovered from water flowing in reverse osmosis sewer pipes. Hydropower is a clean and renewable source of energy. It provides inexpensive electricity and produces no pollution [6]. In a previous study, we equipped our double-stage reverse osmosis system (with a flow rate of 2000 L/h, a reject flow rate of 750 L/h in the first pass and a flow rate of 1600 L/h with a reject flow rate of 600 L/h in the second pass and an operating pressure of 4 bars) with a micro hydro turbine operating on reject osmosis water pressure (Fig. 3).

Our device was able to generate up to 1.6 kWh of electricity per day and 487 kWh per year. This energy was used to power a 100 W light bulb or a LED TV on a daily basis. More interestingly, we were able to eliminate up to 300 kg of CO_2_-eq emissions per year, the equivalent of the CO_2_ emissions generated in a year by one home in an African country [17].

## Extracting nutrients and bio-fertilizers

Recovering nutrients from wastewater to use as soil fertilizers is a challenging task [5, 18]. Struvite crystallization is an effective and sustainable way to achieve both nutrient recovery and the reuse of precipitates [5, 18].

Spent dialysate contains high amounts of phosphorus and nitrogen. These can be recovered as a salt of struvite (MgNH_4_PO_4_⋅6H_2_O), using a chemical crystallization technique by adding magnesium sulfate to the waste fluid. Struvite can be used directly as a plant fertilizer, or as a component in slow-release fertilizer or as raw material for fertilizers [5].

The financial yield of struvite production is interesting: using a tailor-made system for a medium-sized dialysis facility (20 machines and 2 shifts/day), we demonstrated that up to 5 ha of arable land per year can be fertilized. [5]

## Conclusion

The environmental impact of dialysis needs to be addressed. Implementing the pilot experiences reported in this review makes it possible to significantly decrease dialysis waste and produce savings on a large scale. Circular strategies aimed at recovering and reusing dialysis wastewater can reduce the carbon footprint of hemodialysis treatment to about one-third of the current one [6].

The recovery of thermal energy and the production of fertilizers are also promising strategies [5, 6]. Overcoming barriers to these feasible actions should be a priority for the dialysis community; simple practical solutions should be integrated into the indications laid out by Nephrology Societies for the construction of new dialysis units and the green transition of existing ones.

## Data Availability

Data sharing is not applicable to this article as no datasets were generated or analyzed for the current study.
